# Supplementary material for: MEME-LaB: motif analysis in clusters
Source: Bioinformatics. 2013 May 14;29(13):1696–7. doi: 10.1093/bioinformatics/btt248 (PMC3694638; doi:10.1093/bioinformatics/btt248)
Supplement: Supplementary Data [file supp_btt248_Supplementary_Data.zip › Supplementary_Data/Results_Files/which_gene_ids/cluster14_motif1.html]

Cluster 14 Motif 1


Cluster 14 Motif 1 is found in the following promoters

| Gene ID | Strand | Position\* |
| --- | --- | --- |

|  |  |  |
| --- | --- | --- |
| AT1G03090 | - | 462/500 |
| AT1G03410 | - | 485/500 |
| AT1G03680 | - | 397/500 |
| AT1G03910 | + | 424/500 |
| AT1G04850 | - | 156/500 |
| AT1G05150 | + | 59/500 |
| AT1G06200 | + | 266/500 |
| AT1G06390 | + | 224/500 |
| AT1G06960 | + | 251/500 |
| AT1G07210 | + | 128/500 |
| AT1G07830 | + | 373/500 |
| AT1G08390 | - | 347/500 |
| AT1G08660 | + | 97/500 |
| AT1G08780 | + | 391/500 |
| AT1G09150 | + | 170/500 |
| AT1G11430 | - | 354/500 |
| AT1G11440 | + | 3/500 |
| AT1G12620 | + | 256/500 |
| AT1G12850 | + | 123/500 |
| AT1G12920 | - | 124/500 |
| AT1G14140 | - | 262/500 |
| AT1G16210 | + | 134/500 |
| AT1G16260 | - | 311/500 |
| AT1G16810 | - | 111/500 |
| AT1G17490 | + | 141/500 |
| AT1G17560 | + | 181/500 |
| AT1G18160 | + | 299/500 |
| AT1G21690 | - | 293/500 |
| AT1G24490 | + | 462/500 |
| AT1G26570 | + | 391/500 |
| AT1G26830 | + | 291/500 |
| AT1G27400 | + | 168/500 |
| AT1G27510 | + | 385/500 |
| AT1G29890 | - | 458/500 |
| AT1G30610 | - | 298/500 |
| AT1G31940 | + | 436/500 |
| AT1G32750 | + | 87/500 |
| AT1G45130 | + | 255/500 |
| AT1G47290 | + | 25/500 |
| AT1G47310 | - | 237/500 |
| AT1G48380 | - | 438/500 |
| AT1G48650 | - | 221/500 |
| AT1G49970 | + | 23/500 |
| AT1G50320 | + | 104/500 |
| AT1G53645 | - | 83/500 |
| AT1G55750 | - | 358/500 |
| AT1G56090 | + | 139/500 |
| AT1G56440 | - | 158/500 |
| AT1G56610 | - | 332/500 |
| AT1G57870 | - | 424/500 |
| AT1G59760 | + | 401/500 |
| AT1G61100 | + | 354/500 |
| AT1G61170 | - | 159/500 |
| AT1G61620 | + | 392/500 |
| AT1G63670 | - | 298/500 |
| AT1G63990 | + | 144/500 |
| AT1G64550 | + | 308/500 |
| AT1G66920 | - | 274/500 |
| AT1G69510 | + | 71/500 |
| AT1G71350 | + | 431/500 |
| AT1G73090 | + | 328/500 |
| AT1G73230 | + | 347/500 |
| AT1G74250 | + | 360/500 |
| AT1G75980 | + | 316/500 |
| AT1G76280 | - | 128/500 |
| AT1G76300 | - | 319/500 |
| AT1G76400 | + | 270/500 |
| AT1G76670 | + | 391/500 |
| AT1G78150 | + | 400/500 |
| AT1G79150 | + | 214/500 |
| AT1G79380 | + | 74/500 |
| AT1G79390 | + | 236/500 |
| AT1G79430 | - | 55/500 |
| AT1G79850 | + | 191/500 |
| AT1G80070 | - | 273/500 |
| AT1G80410 | - | 159/500 |
| AT1G80560 | + | 482/500 |
| AT1G80680 | + | 320/500 |
| AT1G80940 | + | 382/500 |
| AT2G02470 | + | 235/500 |
| AT2G04270 | + | 44/500 |
| AT2G04890 | + | 118/500 |
| AT2G05120 | + | 289/500 |
| AT2G13540 | + | 481/500 |
| AT2G14045 | - | 290/500 |
| AT2G14080 | + | 318/500 |
| AT2G14910 | - | 289/500 |
| AT2G16090 | + | 410/500 |
| AT2G18400 | + | 359/500 |
| AT2G19830 | + | 257/500 |
| AT2G20990 | - | 20/500 |
| AT2G22310 | + | 86/500 |
| AT2G26500 | + | 459/500 |
| AT2G27285 | + | 6/500 |
| AT2G28310 | + | 314/500 |
| AT2G29550 | - | 487/500 |
| AT2G35480 | + | 262/500 |
| AT2G36490 | + | 303/500 |
| AT2G36930 | + | 69/500 |
| AT2G38630 | - | 101/500 |
| AT2G38770 | - | 68/500 |
| AT2G39795 | + | 273/500 |
| AT2G41520 | - | 486/500 |
| AT2G42120 | + | 293/500 |
| AT2G43130 | + | 285/500 |
| AT2G43770 | + | 463/500 |
| AT2G44150 | + | 477/500 |
| AT2G44180 | - | 234/500 |
| AT2G45030 | - | 71/500 |
| AT2G45520 | - | 345/500 |
| AT2G45720 | + | 144/500 |
| AT2G45770 | + | 183/500 |
| AT2G45990 | + | 113/500 |
| AT2G47980 | + | 476/500 |
| AT3G02180 | - | 291/500 |
| AT3G02220 | + | 219/500 |
| AT3G02860 | + | 245/500 |
| AT3G03150 | + | 221/500 |
| AT3G04890 | + | 442/500 |
| AT3G05070 | + | 100/500 |
| AT3G06930 | - | 307/500 |
| AT3G07930 | + | 389/500 |
| AT3G09150 | - | 80/500 |
| AT3G09980 | + | 30/500 |
| AT3G11730 | - | 95/500 |
| AT3G12010 | - | 131/500 |
| AT3G12380 | + | 293/500 |
| AT3G13060 | - | 215/500 |
| AT3G13222 | + | 141/500 |
| AT3G13226 | - | 93/500 |
| AT3G13690 | - | 437/500 |
| AT3G13860 | + | 229/500 |
| AT3G15410 | + | 60/500 |
| AT3G16940 | - | 55/500 |
| AT3G17090 | - | 276/500 |
| AT3G17330 | - | 34/500 |
| AT3G17465 | - | 338/500 |
| AT3G17650 | - | 128/500 |
| AT3G18165 | - | 474/500 |
| AT3G18190 | - | 486/500 |
| AT3G18990 | - | 487/500 |
| AT3G19340 | + | 464/500 |
| AT3G19400 | + | 440/500 |
| AT3G19670 | - | 392/500 |
| AT3G20050 | + | 190/500 |
| AT3G20790 | - | 43/500 |
| AT3G25150 | - | 294/500 |
| AT3G26618 | - | 24/500 |
| AT3G44600 | + | 229/500 |
| AT3G46960 | + | 120/500 |
| AT3G47590 | - | 441/500 |
| AT3G48430 | - | 350/500 |
| AT3G49180 | + | 324/500 |
| AT3G49190 | - | 174/500 |
| AT3G49490 | - | 196/500 |
| AT3G50960 | - | 104/500 |
| AT3G51310 | - | 220/500 |
| AT3G51720 | + | 374/500 |
| AT3G52905 | - | 141/500 |
| AT3G54010 | + | 347/500 |
| AT3G54190 | + | 319/500 |
| AT3G56760 | - | 216/500 |
| AT3G57150 | + | 160/500 |
| AT3G57700 | + | 370/500 |
| AT3G59650 | + | 316/500 |
| AT3G61050 | + | 318/500 |
| AT3G62140 | - | 382/500 |
| AT3G62800 | - | 251/500 |
| AT3G63030 | + | 241/500 |
| AT4G00270 | - | 449/500 |
| AT4G00730 | + | 388/500 |
| AT4G01210 | + | 80/500 |
| AT4G02640 | + | 438/500 |
| AT4G02720 | - | 146/500 |
| AT4G03000 | - | 440/500 |
| AT4G04720 | + | 430/500 |
| AT4G09150 | - | 209/500 |
| AT4G11260 | - | 329/500 |
| AT4G14605 | - | 106/500 |
| AT4G15520 | + | 159/500 |
| AT4G16420 | - | 393/500 |
| AT4G17330 | - | 257/500 |
| AT4G17370 | + | 395/500 |
| AT4G18700 | + | 409/500 |
| AT4G19003 | - | 178/500 |
| AT4G22290 | + | 227/500 |
| AT4G24940 | - | 443/500 |
| AT4G25360 | - | 272/500 |
| AT4G25960 | - | 96/500 |
| AT4G26630 | + | 63/500 |
| AT4G27120 | - | 214/500 |
| AT4G27710 | - | 247/500 |
| AT4G31420 | + | 236/500 |
| AT4G31460 | - | 338/500 |
| AT4G31560 | - | 257/500 |
| AT4G31930 | + | 152/500 |
| AT4G32120 | - | 230/500 |
| AT4G32590 | - | 91/500 |
| AT4G32605 | + | 28/500 |
| AT4G32910 | + | 111/500 |
| AT4G33250 | + | 114/500 |
| AT4G34140 | - | 171/500 |
| AT4G35540 | - | 402/500 |
| AT5G02150 | + | 118/500 |
| AT5G02620 | + | 353/500 |
| AT5G03100 | + | 210/500 |
| AT5G03520 | - | 223/500 |
| AT5G03880 | + | 51/500 |
| AT5G05460 | - | 249/500 |
| AT5G05610 | - | 359/500 |
| AT5G05980 | - | 442/500 |
| AT5G07090 | + | 355/500 |
| AT5G07340 | - | 149/500 |
| AT5G08280 | - | 304/500 |
| AT5G09450 | + | 248/500 |
| AT5G09740 | - | 120/500 |
| AT5G10870 | - | 475/500 |
| AT5G13950 | + | 389/500 |
| AT5G13960 | + | 68/500 |
| AT5G14930 | - | 457/500 |
| AT5G15610 | + | 35/500 |
| AT5G15880 | + | 488/500 |
| AT5G16130 | - | 148/500 |
| AT5G16780 | - | 135/500 |
| AT5G16880 | + | 368/500 |
| AT5G17880 | + | 197/500 |
| AT5G18410 | - | 270/500 |
| AT5G18580 | + | 454/500 |
| AT5G19010 | + | 417/500 |
| AT5G19450 | - | 15/500 |
| AT5G19680 | - | 296/500 |
| AT5G19990 | + | 234/500 |
| AT5G20840 | - | 221/500 |
| AT5G20885 | - | 42/500 |
| AT5G20890 | - | 292/500 |
| AT5G20980 | + | 88/500 |
| AT5G22400 | - | 331/500 |
| AT5G23080 | - | 470/500 |
| AT5G23440 | + | 482/500 |
| AT5G23890 | - | 324/500 |
| AT5G41340 | + | 396/500 |
| AT5G41770 | - | 56/500 |
| AT5G42190 | + | 434/500 |
| AT5G43740 | + | 397/500 |
| AT5G44580 | + | 40/500 |
| AT5G45500 | - | 188/500 |
| AT5G46210 | - | 311/500 |
| AT5G47320 | - | 203/500 |
| AT5G47490 | - | 108/500 |
| AT5G48520 | - | 304/500 |
| AT5G48990 | - | 177/500 |
| AT5G51280 | - | 394/500 |
| AT5G51940 | + | 314/500 |
| AT5G52440 | + | 273/500 |
| AT5G52520 | + | 89/500 |
| AT5G52820 | + | 448/500 |
| AT5G55140 | - | 37/500 |
| AT5G55640 | + | 300/500 |
| AT5G56040 | - | 255/500 |
| AT5G56130 | - | 312/500 |
| AT5G56140 | + | 405/500 |
| AT5G56280 | + | 28/500 |
| AT5G56360 | + | 272/500 |
| AT5G58030 | + | 276/500 |
| AT5G58100 | - | 489/500 |
| AT5G58590 | + | 15/500 |
| AT5G59210 | - | 295/500 |
| AT5G60920 | + | 1/500 |
| AT5G60960 | + | 35/500 |
| AT5G62980 | - | 99/500 |
| AT5G64600 | + | 445/500 |
| AT5G64830 | + | 91/500 |
| AT5G65180 | - | 319/500 |
| AT5G65880 | - | 386/500 |
| AT5G66030 | + | 85/500 |
| AT5G66820 | + | 255/500 |

**\*** Overlapping sequences are merged to avoid redundancy. As a result some sequences in the final set may be longer than the specified maximum.

Distribution

+

-

Position values represent number of bases from the 5 prime end of the sequence provided to MEME and the length of that sequence. It is these figures expressed as proportions that are used for statistical analysis.

Probability value of comparison of these values to a linear distribution, p = 0.17

The motif is found 151 times on the + stand and 125 times on the - strand.

Probability value that it is evenly distributed between the two strands, p = 0.066

Move the mouse pointer over the gene ids to highlight the corresponding marker
